# Case Report: Identification of a novel mutation, c.1067T > A, in the *SERPING1* gene in a Chinese male with type 1 hereditary angioedema

**DOI:** 10.3389/falgy.2025.1554940

**Published:** 2025-04-29

**Authors:** Wenjin Du, Ke Yang, Qiuxing Zhang, Xianghua Lin, Wenchao Zhang, Weili Guo, Zhaoji Meng, Siqin Wang

**Affiliations:** ^1^ Department of Allergy, Henan Provincial People’s Hospital, Zhengzhou, China; ^2^ Department of Allergy, People’s Hospital of Zhengzhou University, Zhengzhou, China; ^3^ Department of Allergy, People’s Hospital of Henan University, Zhengzhou, China; ^4^ Henan Key Laboratory of Genetic Diseases and Functional Genomics, People’s Hospital of Henan University, Zhengzhou, China; ^5^ Medical Genetics Institute, Zhengzhou University People’s Hospital, Zhengzhou, China

**Keywords:** *SERPING1* gene, hereditary angioedema (HAE), mutation, case report, C1 inhibitor (C1INH)

## Abstract

Hereditary angioedema (HAE) is a rare autosomal dominant genetic disorder characterized by recurrent, unpredictable episodes of angioedema that commonly involve the face, limbs, respiratory tract, and gastrointestinal tract. Clinical presentations vary substantially among individuals, increasing the likelihood of misdiagnosis or missed diagnosis. In severe cases, if not properly managed, laryngeal edema can result in asphyxiation or even death. Here, we report a Chinese male patient who experienced recurrent limb swelling and abdominal pain. Laboratory tests revealed low levels of complement C4 and C1 inhibitors, along with impaired C1 inhibitor function. Genomic DNA extracted from peripheral blood samples underwent PCR amplification and Sanger sequencing, which identified a *de novo* heterozygous mutation in the *SERPING1* gene at chr11:57379227, confirming a novel missense mutation NM_000062.c.1067T > A (p.V356E). Ultimately, the patient was diagnosed with HAE-C1INH-Type1 and successfully protected from recurrent attacks through subcutaneous administration of lanadelumab.

## 1 Introduction

Hereditary angioedema (HAE) is a rare autosomal dominant disorder affecting approximately 1/50,000 people globally (1). It is characterized by unpredictable and painful swelling, typically involving the face, oropharynx, abdomen, extremities, and genitals. Swelling episodes often result in significant functional impairment, reduced quality of life, and potentially fatal outcomes in case of laryngeal attacks (1, 2). HAE is classified into two categories: HAE due to C1-esterase inhibitor deficiency (HAE-C1INH) and HAE with normal C1INH. HAE-C1INH is further divided into types 1 and 2, based on the deficiency or dysfunction of circulating C1INH protein caused by inherited or spontaneous mutations in the *SERPING1* gene. These mutations lead to uncontrolled activation of factor XII and plasma kallikrein, resulting in excessive bradykinin production and recurrent episodes of subcutaneous or submucosal swelling (3–5). While more than 700 *SERPING1* variants have been identified worldwide (6), only about 60 have been reported in the Chinese population (7). The *SERPING1* gene, the known pathogenic gene for HAE, is located on chromosome 11 (q11_q13.1) and consists of eight exons and seven introns. Previous studies indicate that approximately 75% of HAE patients (types 1 or 2) exhibit an autosomal dominant inheritance pattern, resulting in a 50% chance of passing the disease to their offspring (3). In addition, approximately 20%–25% of patients represent *de novo* cases within a family (8). Research has underscored the importance of parental genetic testing for all patients, regardless of whether the parents are affected, and highlighted the implications of gonosomal mosaicism for genetic counseling (9). In this report, we present a novel *SERPING1* gene mutation that caused type 1 HAE.

## 2 Case report

**Figure 1:**
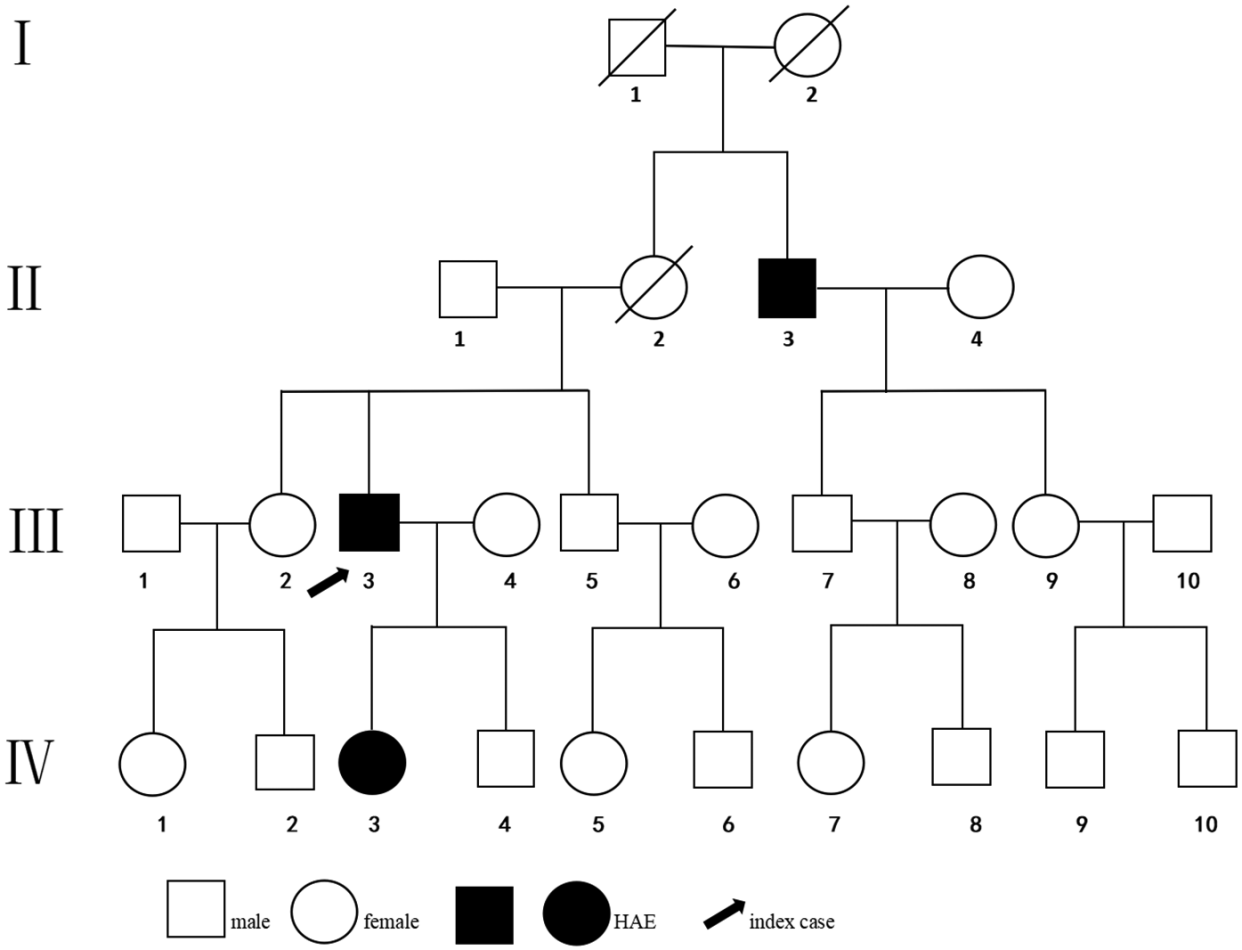
Pedigree of the family with HAE. Circles indicate females, squares indicate males, black-filled symbols indicate affected individuals, the arrow indicates the index patient, and a slash indicates a deceased individual.

**Figure 2:**
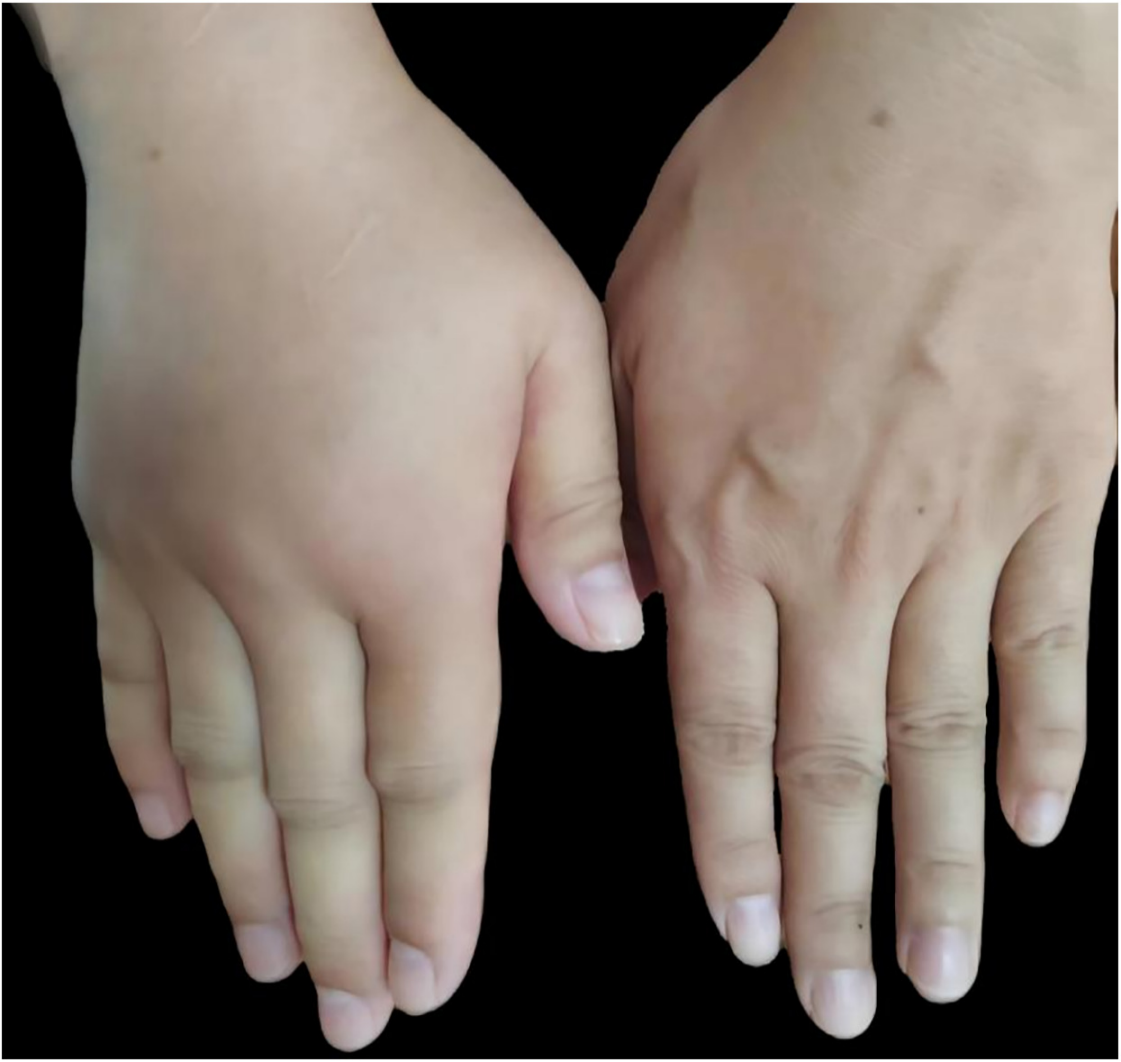
Clinical image. Non-pitting edema is visible in the index patient’s right hand.

The index patient (Figure 1), a 34-year-old man, began experiencing localized edema of the limbs, skin, and buttocks 8 years ago. Each episode varied in severity and, regardless of whether the patient received medical treatment, every swelling episode resolved completely on its own within 2–3 days. Subsequently, these symptoms recurred irregularly. Over the previous 6 months, the episodes became more frequent (occurring more than three times in a month), often accompanied by abdominal pain, with some attacks triggered by fatigue. The index patient had no prior history of surgery and special medication. A family history inquiry revealed that his mother had passed away due to “laryngeal edema,” his elder sister experienced buttock swelling after prolonged sitting, and his uncle suffered episodic abdominal pain and unilateral upper-limb swelling, thus, both had similar histories of edema. At presentation, his vital signs were within normal limits. On physical examination, the index patient exhibited non-pitting edema in the right hand (Figure 2). Laboratory tests showed that complete blood count, D-dimer, PCT, CRP, HP, 25(OH)D, immunoglobulin A/G/M, C3, C1q, rheumatoid factors, ANA + ENA, ANCA qualitative, total IgE, and specific IgE (the specific IgE against *Dermatophagoides farinae*, *Dermatophagoides pteronyssinus*, *Aspergillus fumigatus*, *Alternaria alternata*, *Ambrosia artemisiifolia*, *Artemisia sieversiana*, *Humulus scandens*, *Platanus acerifolia*, cat hair, dog hair, protein, milk, peanuts, fish, wheat, and soybeans) were all within normal range. Tests for HBsAg, HBeAb, and HBcAb were positive (the patient denied any past history of hepatitis and refused to undergo quantitative HBV-DNA testing).

**Figure 3:**
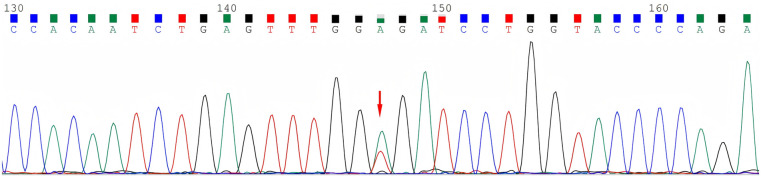
Sequencing map. Heterozygous missense mutation of the *SERPING1* gene (NM_000062) c.1067T > A in the patients.

Serum C4 levels, C1INH concentrations, and functional assays were assessed for this family (Table 1). The index patient's C4 level was 0.02 g/L (reference range: 0.1–0.4 g/L), C1INH concentration was 0.07 g/L (reference range: 0.21–0.39 g/L), and C1INH functional activity was 4.3% (reference range: ≥68.0%). In addition, his 5-year-old asymptomatic daughter and his uncle both exhibited reduced C4 levels, with their C1INH concentrations and functional activities measuring below 50% of the normal lower limit, consistent with a diagnosis of type 1 HAE. Genomic DNA was extracted from peripheral blood samples, and the coding region of the *SERPING1* gene (NM_000062) was analyzed using PCR amplification and Sanger sequencing. A *de novo* heterozygous missense mutation, NM_000062: c.1067T > A (p.V356E), was identified in the *SERPING1* gene at chr11:57379227. Sequence analysis confirmed a heterozygous missense variant in exon 7 of the *SERPING1* gene, resulting in an amino acid substitution at position 356 to change from valine to glutamic acid. The same *SERPING1* gene mutation was subsequently identified in the patient's daughter (IV3) and uncle (II3) (Figure 3). Due to the family members’ refusal to provide blood samples for genetic testing, we were unable to verify the sequencing of the suspected pathogenic mutations in individuals with normal serum C4 and C1INH concentrations and function.

**Table 1:** The C4 and C1INH serum level results.

Subject	Laboratory test (normal ranges)		
	C4 (0.10–0.40 g/L)	C1INH (0.21–0.39 g/L)	C1INH function (≥68.0%)
Ⅱ1	0.19	0.27	87.8
Ⅱ3	0.09	0.05	2.5
Ⅱ4	0.31	0.36	>93
Ⅲ1	0.29	0.23	91.2
Ⅲ2	0.18	0.34	79.7
Ⅲ3	0.02	0.07	4.3
Ⅲ4	0.23	0.34	93.8
Ⅲ5	0.33	0.29	82.9
Ⅲ6	0.38	0.33	>93.0
Ⅲ7	0.24	0.25	84.9
Ⅲ8	0.34	0.31	>93.0
Ⅲ9	0.38	0.33	72.6
Ⅲ10	0.25	0.28	79.3
Ⅳ1	0.24	0.33	>93.0
Ⅳ2	0.37	0.38	89.5
Ⅳ3	0.08	0.08	21.5
Ⅳ4	0.33	0.24	86.7
Ⅳ5	0.27	0.35	>93.0
Ⅳ6	0.31	0.36	>93.0
Ⅳ7	0.33	0.29	71.3
Ⅳ8	0.17	0.23	89.5
Ⅳ9	0.38	0.20	>93.0
Ⅳ10	0.19	0.26	89.4

According to VarSome (https://varsome.com), the *SERPING1* gene mutation c.1067T > A occurs in a functional domain (PM1_Moderate). The REVEL software predicts a score of 0.930 for this mutation, supporting evidence for pathogenicity (PP3_Strong). This variant is not listed in the ClinVar (https://www.ncbi.nlm.nih.gov/clinvar/), HGMD (http://www.hgmd.cf.ac.uk/), or the Leiden Open Variation Database (LOVD, https://databases.lovd.nl/shared/variants/SERPING1), and no carrier frequency data were available for East Asian populations in the gnomAD_exome (http://gnomad.broadinstitute.org) (PM2_Supporting). Based on the American College of Medical Genetics and Genomics (ACMG) standards and guidelines for variant classification, this variant can be categorized as “likely pathogenic” (PP3_Strong + PM1_Moderate + PM2_Supporting). While we predicted this mutation to be pathogenic using *in silico* modeling tools, supported these predictions by measuring serum C4 and C1INH concentrations and functions within this family, and considered the clinical characteristics of the patients, the lack of additional family genetic sequencing to exclude asymptomatic carriers and perform segregation studies imposes certain limitations on our study.

## 3 Discussion

We have identified a previously unreported mutation, c.1067T > A, in the *SERPING1* gene, with a REVEL score of 0.930, suggesting that c.1067T > A may impact protein function. This newly discovered mutation is likely the underlying cause of the disease in this family. We recommend genetic testing for HAE patients and their relatives to enable the early identification of mutation carriers, particularly those who are asymptomatic or exhibit atypical symptoms, ensuring timely and targeted prevention and treatment. Such testing can enhance patient quality of life, lower mortality rates, and provide molecular-level diagnostic insights for precise diagnosis and genetic counseling.

In China, the average age of onset for HAE is approximately 21.25 years, with approximately 75% of patients experiencing their first episode between the ages of 10 and 30. However, the interval between onset and definitive diagnosis is approximately 12.64 years (10). Several factors may contribute to the generally later onset of HAE observed in Chinese patients. These include limited disease awareness and diagnostic capabilities, which may inflate the recorded age of onset, and genetic or environmental variations that could influence clinical phenotypes. Further research and epidemiological studies are needed to clarify these underlying factors. HAE presents with a wide range of clinical manifestations, from asymptomatic cases to life-threatening episodes, with the severity, frequency, and affected areas varying significantly, even within the same family. Edema can occur in any part of the body, most commonly affecting the limbs, face, genitals, respiratory tract, and gastrointestinal mucosa. Upper airway edema is particularly concerning, as laryngeal involvement can progress rapidly, leading to respiratory distress or asphyxiation; without prompt treatment, this can be fatal. Gastrointestinal involvement can cause severe abdominal pain, nausea, and vomiting, which are often mistaken for acute abdomen and may result in unnecessary surgical interventions. Although HAE edema episodes typically resolve on their own, 56.86% of Chinese HAE patients experience laryngeal edema, and 11.39% die from asphyxia caused by laryngeal swelling (10, 11). Consequently, early diagnosis and appropriate treatment are essential for improving outcomes and enhancing the overall quality of life for individuals affected by this serious and potentially disabling disease. According to current guidelines (3), the primary goal of HAE management is to normalize patients’ lives and achieve total disease control (zero episodes). Treatment approaches are generally categorized into two types: on-demand therapy [icatibant, ecalantide, recombinant human C1 inhibitor (rhC1INH), and plasma-derived C1 inhibitor (pd-C1INH)] and prophylactic therapy (lanadelumab, berotralstat, and pd-C1INH). Unfortunately, ecalantide and berotralstat are not yet approved for marketing in China.

Due to the patient's frequent episodes of edema over the previous 6 months, accompanied by abdominal pain, we initiated long-term prophylactic treatment with subcutaneous lanadelumab (300 mg every 2 weeks) on 20 April 2024. Over nearly 10 months of prophylactic treatment with lanadelumab, the patient has experienced only six mild edema episodes, occurring on 25 May (left hand), 23 June (right foot), 26 August (left foot with pain), 31 October (right shoulder joint), and 3 December 2024 (left hand) and 27 January 2025 (right hand), each resolving spontaneously without requiring on-demand therapy. The most recent episode was suspected to be related to compression of the right hand and fully resolved within 1 day. In addition, the patient's angioedema control test (AECT) scores consistently remained above 11 during this 10-month period, reaching 13 on 10 March 2025. These clinical outcomes indicate effective disease management, as the mild and brief breakthrough episodes did not meaningfully impact the patient's quality of life. In accordance with guideline recommendations (3) and based on the patient's current level of disease control, we are continuing prophylactic treatment at 300 mg every 2 weeks.

## Data Availability

The datasets presented in this study can be found in online repositories. The names of the repository/repositories and accession number(s) can be found in the article/Supplementary Material.
